# Conjoined nerve root in a patient with lumbar disc herniation accompanied by a lumbosacral spine anomaly: a case report

**DOI:** 10.1186/s13256-022-03749-1

**Published:** 2023-02-24

**Authors:** Hiroshi Kuroki, Takuya Nagai

**Affiliations:** 1 Department of Orthopaedic Surgery, National Hospital Organization Miyazaki Higashi Hospital, 4374-1 Tayoshi Ooaza, Miyazaki, 880-0911 Japan; 2 Department of Orthopaedic Surgery, University of Miyazaki Faculty of Medicine, Miyazaki, Japan

**Keywords:** Conjoined nerve root, Nerve root anomaly, Anomalous nerve root, Lumbosacral spine anomaly, Spina bifida occulta, Lumbar disc herniation, Microscopic lumbar discectomy, Magnetic resonance imaging, MR neurography, Case report

## Abstract

**Background:**

A nerve root anomaly, typified by a conjoined nerve root, is a rare finding. Conjoined nerve root anomalies are easily missed even in preoperative advanced imaging modalities, which can be potentially troublesome during and after surgery. In this report, we present a case of conjoined right L5–S1 nerve root in a patient with lumbar disc herniation, accompanied by spina bifida occulta, which was undiagnosed on preoperative imaging studies.

**Case report:**

A 55-year-old Asian (Japanese) woman presented with low back pain and right leg radiating pain due to lumbar disc herniation at the right L5/S1. Physical examination revealed a positive Lasègue sign and the range of the straight leg raising test was 20° on the right side. The right patellar tendon reflex was normal; however, the right ankle jerk reflex disappeared. Although no obvious hypoesthesia was noted, mild muscle weakness (4/5) was observed in the right leg on the manual muscle test. We planned the lumbar discectomy under a microscope. During surgery, the conjoined right L5–S1 nerve root, which was compressed by herniated nucleus pulposus, was encountered. Although it was very thick and less mobile, some pieces of herniated nucleus pulposus could be removed piece by piece from the axillary part. After sequential decompressive procedures, the tightness of the conjoined right L5–S1 nerve root decreased but its mobility did not improve much. The laterality of the thickness and exit angle of the conjoined right L5–S1 nerve root was retrospectively confirmed on T2 coronal magnetic resonance images and magnetic resonance neurography. Postoperatively, right leg pain was immediately alleviated and complete improvement of muscle weakness was achieved 1 week later (5/5).

**Conclusions:**

Magnetic resonance neurography is extremely useful for the accurate diagnosis of anomalous nerve roots because of clear visualization of the neural tissue. Discectomy under a microscope, which enables magnified three-dimensional observation of the surgical field, must provide a valid and safe procedure to achieve not only secure resection of herniated discs but also adequate exposure of anomalous nerve roots.

## Introduction

The lumbar nerve root, which is surrounded by a root sleeve extension of the dura mater, normally runs from the medial to lateral direction beneath the corresponding vertebral pedicle, exiting through the respective intervertebral foramen [1]. However, a nerve root anomaly (NRA), typified by a conjoined nerve root (CNR), is a rare finding. NRAs likely result from aberrant migration of the involved roots during embryological development [2]. A CNR is defined as two adjacent nerve roots that share a common dural envelope at some time during their course from the thecal sac [3]. CNR anomalies are easily missed, even in preoperative advanced imaging modalities, which can be potentially troublesome during and after surgery [4].

Lumbosacral NRAs were first described by Zagnoni [5] in 1949. Subsequently, Ethelberg *et al*. [6] reported that four cases (0.34%) of significant NRAs were found in 1162 patients who underwent lumbar laminectomy in 1952. In 1962, Cannon *et al*. [2] encountered an NRA in 2% of lumbar surgery cases, and categorized them into three types: conjoined roots, anastomotic roots, and transverse roots. Similar case reports have sporadically appeared in the literature.

The prevalence of lumbosacral NRAs has varied widely since early reports, as previously shown. The anatomical dissections of 100 cadaveric specimens by Kadish *et al*. [7] revealed 23 NRAs in 14 lumbosacral spines (14%). Postacchini *et al*. [8] found 46 lumbosacral NRAs in a series of 2123 patients who underwent myelography for low back and radicular pain (2%). Haijiao *et al*. [9] detected 65 cases of congenital NRAs in a retrospective analysis of 376 patients who underwent magnetic resonance imaging (MRI) of the lumbar spine for low back pain and/or radicular pain (17.3%). Artico *et al*. [10] identified three patients with lumbosacral CNRs, representing 0.25% of 1200 patients who underwent lumbosacral CT/MRI procedures. White *et al*. [11] detected that in their 25-year series of 4726 disc operations, 63 CNRs were identified unequivocally, with an incidence of 1.3%. Scuderi *et al*. [3] reported that 4 in 80 patients were intraoperatively found to have evidence of a CNR (5%). In a more recent report of a cohort undergoing lumbar microdiscectomy by Lotan *et al*. [12], the incidence of CNR identified during surgery was 5.8%.

In this report, we present a case of conjoined L5–S1 nerve root in a patient with lumbar disc herniation, accompanied by spina bifida occulta, which was undiagnosed on preoperative imaging studies.

## Case report

A 55-year-old Asian (Japanese) woman presented with low back pain and right leg radiating pain without any apparent cause. She received conservative treatment, such as pain killer administration and lumbar root block injection under the diagnosis of lumbar disc herniation at the right L5/S. However, her symptoms gradually worsened until she could not walk because of severe right leg pain and mild muscle weakness. She then referred to our hospital for surgical treatment.

Physical examination revealed a positive Lasègue sign and the range of the straight leg raising test was 20° on the right side. The right patellar tendon reflex was normal; however, the right ankle jerk reflex absent. On the manual muscle test (MMT), mild muscle weakness (4/5) was observed in the right tibialis anterior (TA), peroneal longus et brevis (PL et B), extensor hallucis longus (EHL), flexor hallucis longus (FHL), and triceps surae (TS). Although there was occasional mild numbness in the right lower leg, no obvious hypoesthesia was noted in the definitive area.

**Fig. 1:**
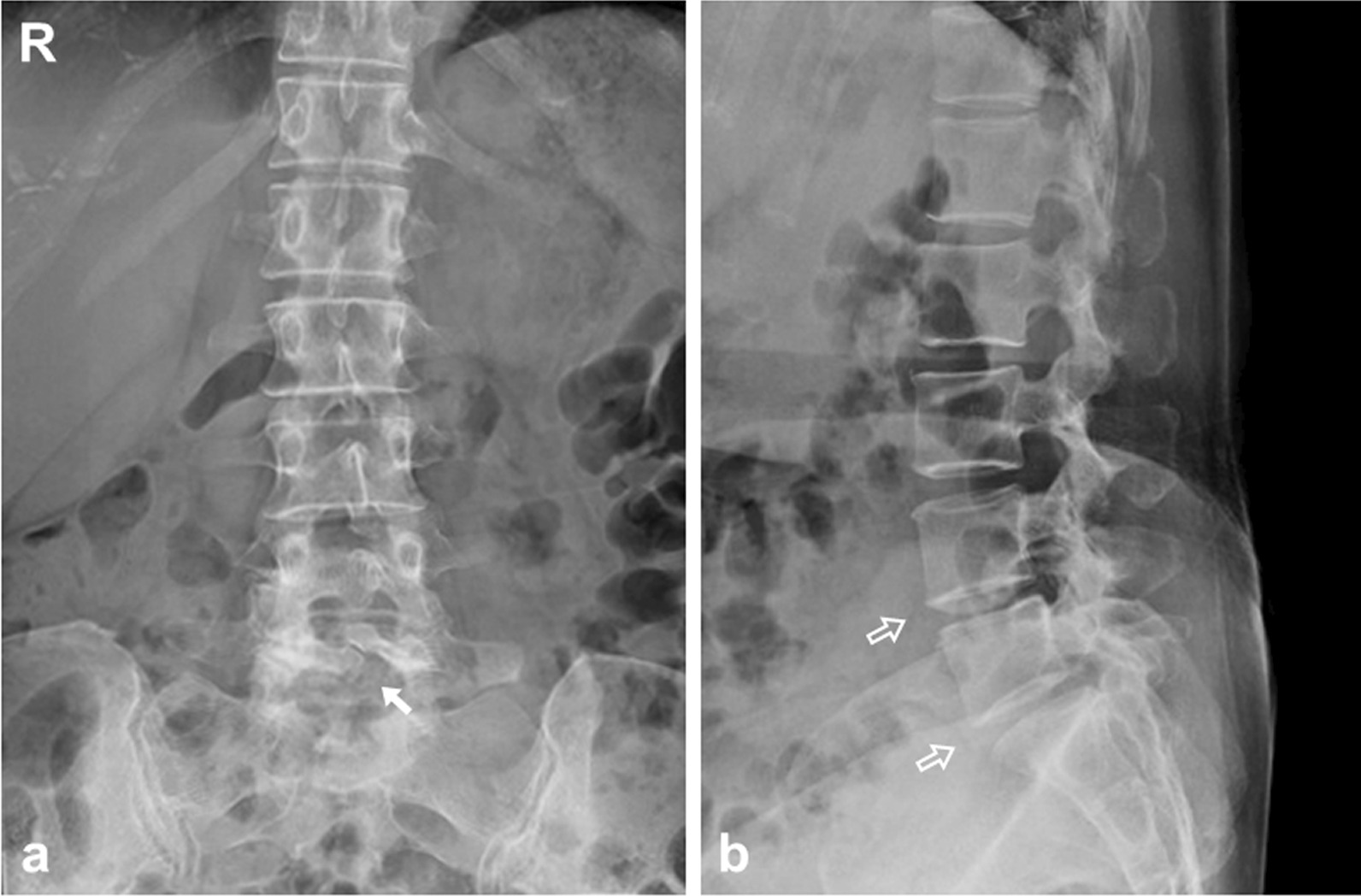
Preoperative plain radiographs. Spina bifida occulta at the L5 neural arch was detected in the anteroposterior direction (white arrow), and slight disc space narrowing at the L4/5 and L5/S intervertebral disc levels was seen in the lateral direction (open arrows). **a** a-p view. **b** lateral view.

**Fig. 2:**
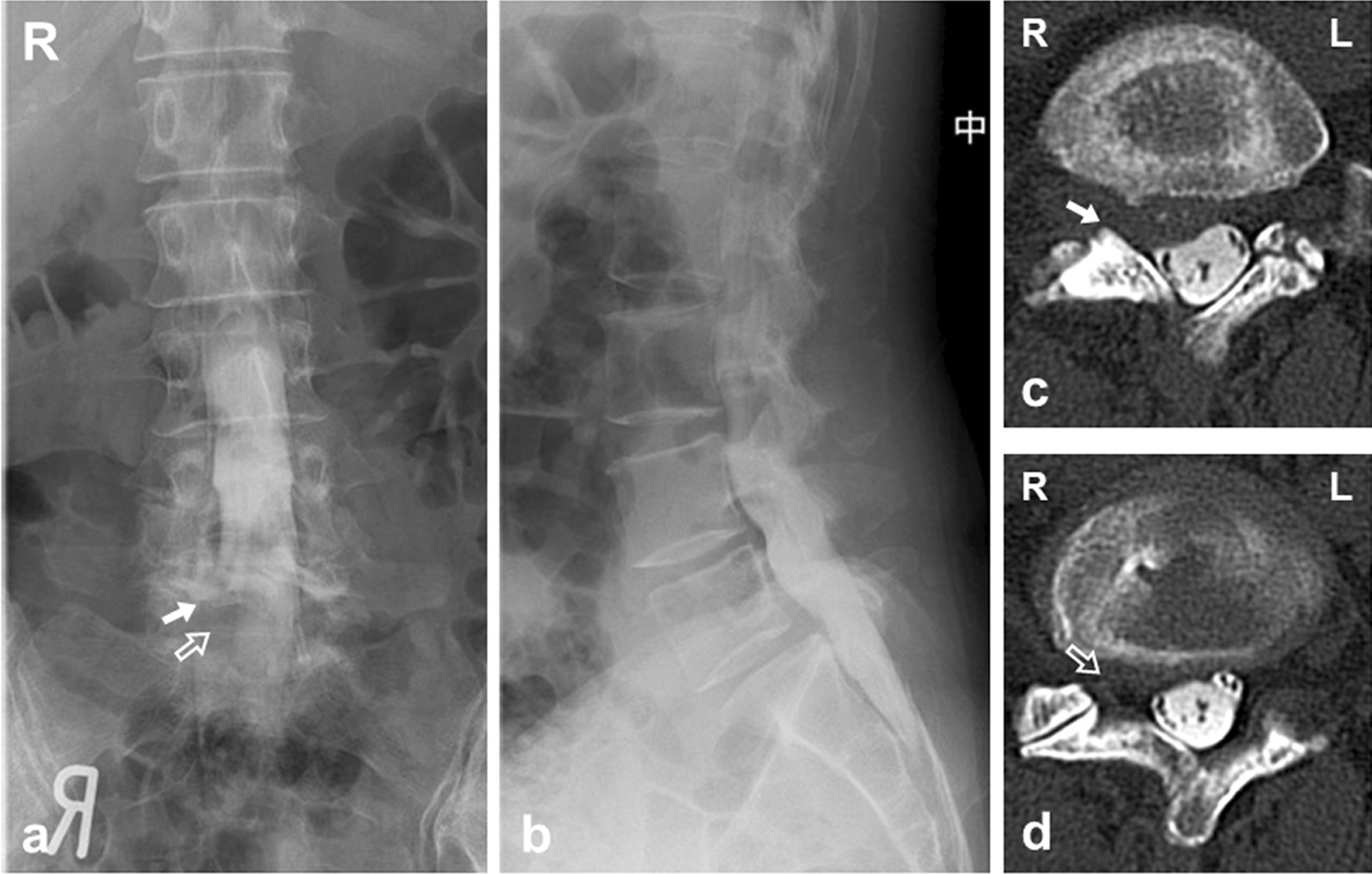
Myelography and CT following myelography (CTM). The defect in the right L5 and S1 nerve roots was shown (white arrow: right L5 root, open arrow: right S1 nerve root). **a** a-p view. **b** lateral view. **c**, **d** axial view of CTM

**Fig. 3:**
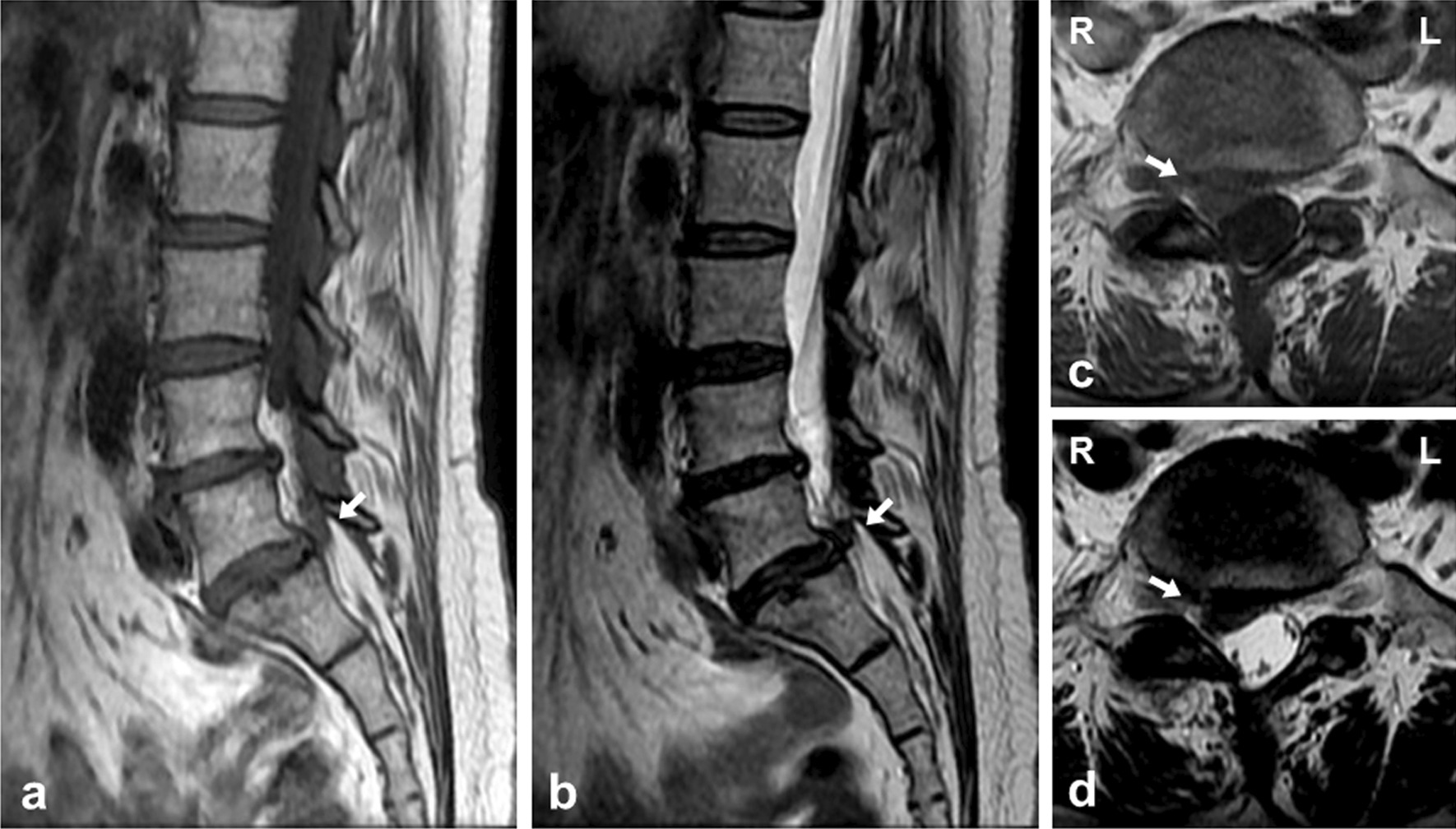
MRI. Herniated nucleus pulposus on the right side at the L5/S1 intervertebral disc level was revealed (white arrows). **a** T1 parasagittal view. **b** T2 parasagittal view. **c** T1 axial view. **d** T2 axial view

On plain radiograph, spina bifida occulta at the L5 neural arch was detected in the anteroposterior direction, and slight disc space narrowing at the L4/5 and L5/S intervertebral disc levels was seen in the lateral direction (Fig. 1). Myelography and computed tomography (CT) following myelography (CTM) showed a defect in the right L5 and S1 nerve roots (Fig. 2). Magnetic resonance (MR) images revealed a herniated nucleus pulposus (HNP) on the right side at the L5/S1 intervertebral disc level (Fig. 3). Our preoperative diagnosis was right S1 radiculopathy originating from a usual HNP that compresses the right S1 nerve root in accordance with the findings of L5 radiculopathy suspected by muscle weakness of the TA, EHL, and PL et B.

**Fig. 4:**
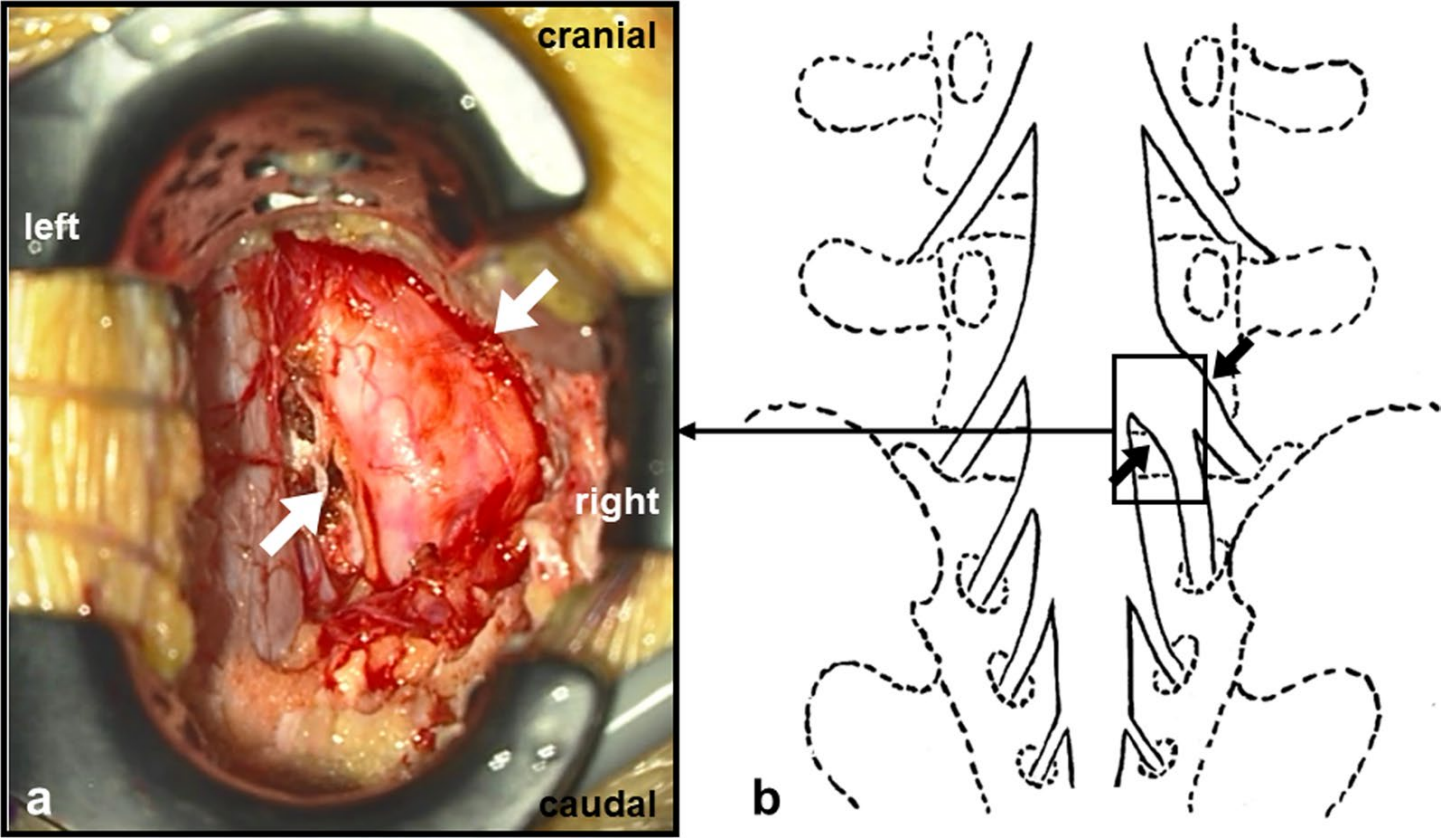
Intraoperative image and schema. The thickened and less mobile conjoined right L5–S1 nerve root was indicated at the L5/S1 intervertebral disc level (white and black arrows). **a** image. **b** schema

**Fig. 5:**
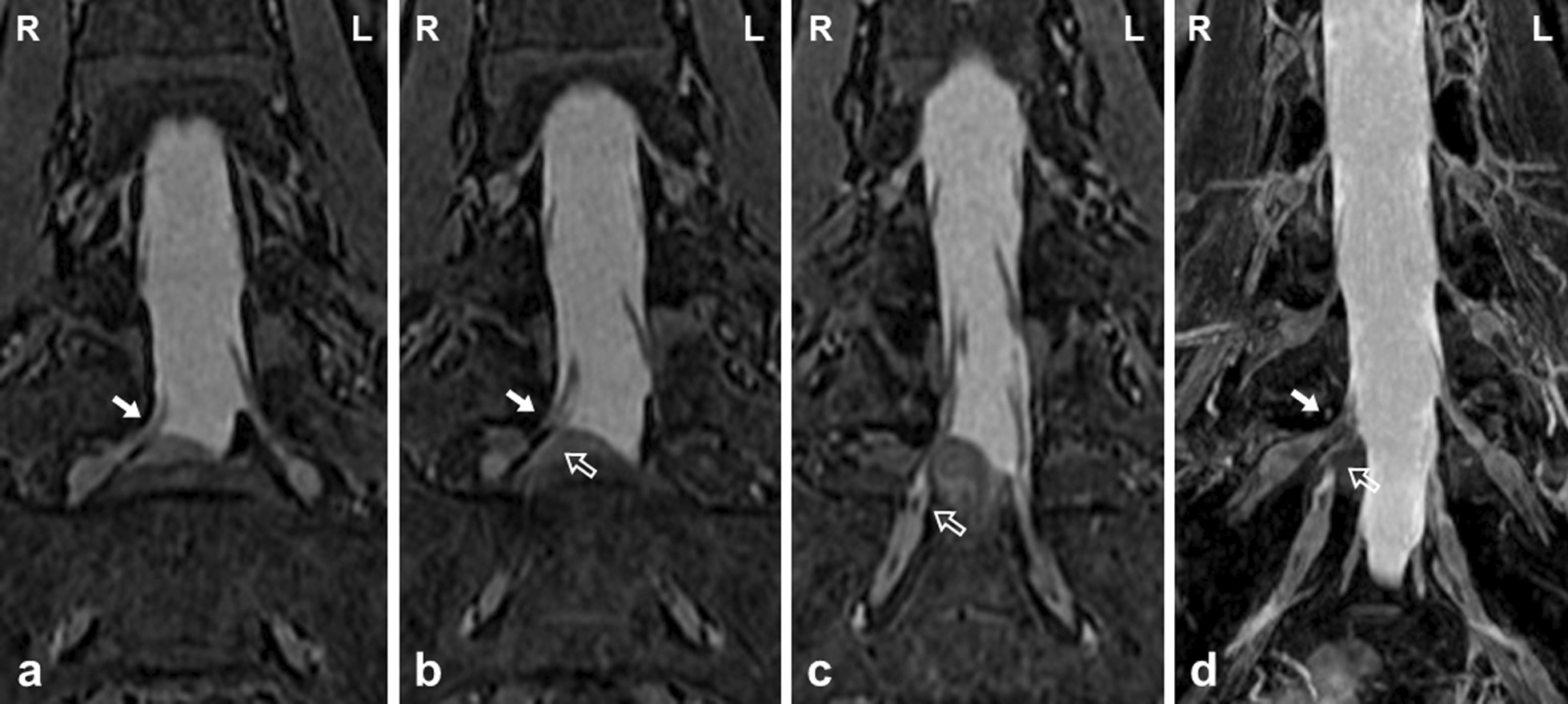
MRI. The laterality of the thickness and exit angle of the conjoined right L5–S1 nerve root was retrospectively confirmed (white arrow: right L5 root; open arrow: right S1 nerve root). **a** T2 coronal view at the L5 nerve root. **b** T2 coronal view at the L5/S1 nerve roots. **c** T2 coronal view at the S1 nerve root. **d** MR neurography

We planned lumbar discectomy under a microscope. First, wide fenestration at the right L5/S interlaminar space was performed to identify the right S1 nerve root that was compressed by HNP. However, an anomaly of the conjoined right L5–S1 nerve root was observed. There was no adhesion between the yellow ligament and neural tissue. The epidural space was filled with sufficient adipose tissue. An HNP extruding from the right L5/S intervertebral disc space was confirmed beneath the conjoined right L5–S1 nerve root, which was severely compressed and shifted backward. Mild adhesion between the conjoined right L5–S1 nerve root and capsule of the HNP was observed. Next, to ensure safe discectomy, the area of the fenestration was enlarged while assuring that the facet joint was not disrupted. Although the conjoined right L5–S1 nerve root was very thick and less mobile, some pieces of HNP could be removed piece by piece from the axillary part of the conjoined right L5–S1 nerve root. After these decompressive procedures, the tightness of the conjoined right L5–S1 nerve root decreased, but its mobility did not improve much (Fig. 4). The laterality of the thickness and exit angle of the conjoined right L5–S1 nerve root was retrospectively confirmed on preoperative T2 coronal MR images and MR neurography (Fig. 5).

**Fig. 6:**
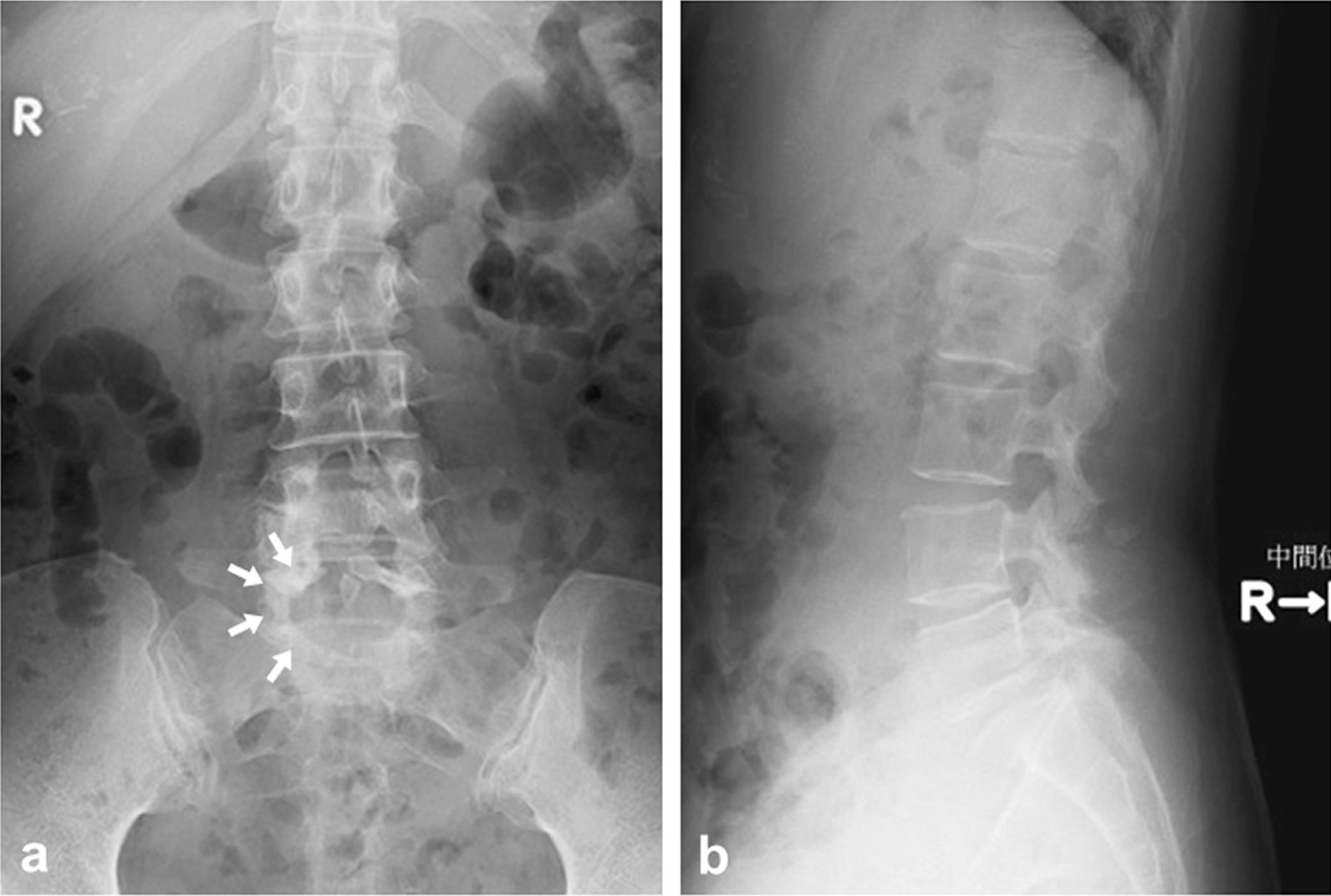
Plain radiographs at 6 months after surgery. Wide fenestration at the right L5/S1 interlaminar space was performed (white arrows). **a** a-p view. **b** lateral view

Postoperatively, right leg pain was immediately alleviated and complete improvement of muscle weakness was achieved 1 week later (5/5). At 6 months after surgery, she complained of mild pain at the back and the bilateral thighs, similar to myalgia but was able to walk without any support (Fig. 6).

## Discussion

**Fig. 7:**
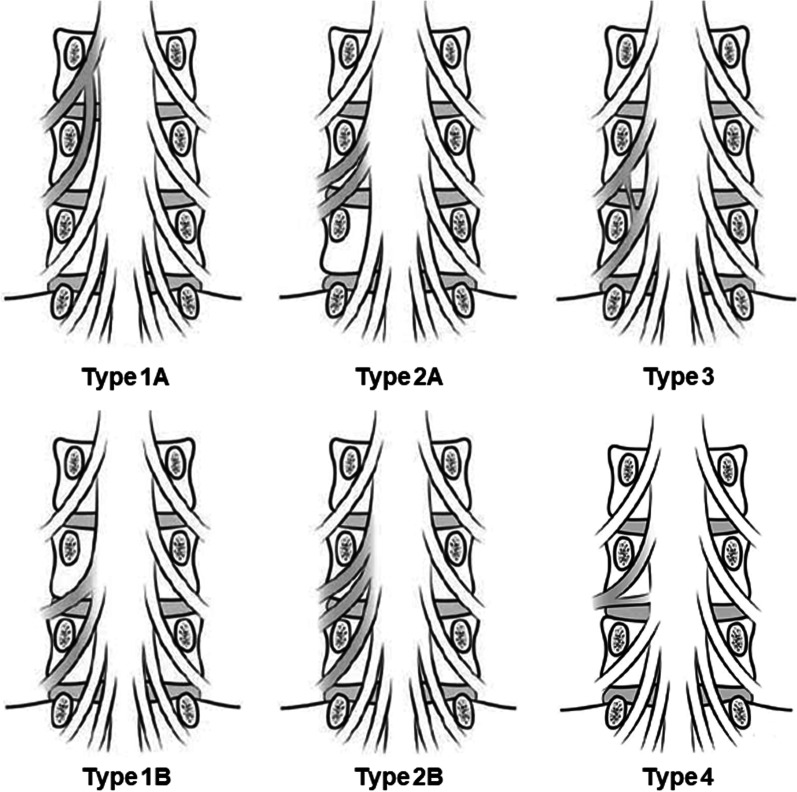
Classification of nerve root anomalies (NRAs) by Neidre and Macnab including a fourth entry of NRA described by Burke *et al*. Type 1 anomalies include those of conjoined nerve roots arising from a common dural sheath. Type 2 anomalies are most common, where two nerve roots exit through the same foramen. Type 3 anomalies are those in which adjacent nerve roots are connected in the form of a transverse or vertical anastomosis. Type 4 anomaly is a confluent nerve root with two contributions arising from the thecal sac coalescing to form a single nerve root that exits via the intervertebral foramen

Neidre and Macnab [13] proposed the classification of NRA based on Cannon’s classification and considering their own cases. They divided NRA into three types and subtypes according to the way the nerve roots exit the dura and the spinal canal. However, more recently, Burke *et al*. [14] suggested a fourth entry where a confluent nerve root exits the foramen with two contributions from two adjacent nerve roots arising separately from the thecal sac (Fig. 7).

There are some atypical clinical findings in patients with disc herniation with CNRs. CNRs are rarely associated with other spinal anomalies, including defects in the neural arch, spondylolysis or spondylolisthesis, spina bifida occulta, or other bony anomalies [2]. Jokhi *et al*. [15] reported a case of a conjoined L4–5 nerve root accompanied by spina bifida at the L5 level with severe lumbar canal stenosis. In addition, Okuwaki *et al*. [16] introduced a case of conjoined L5–S1 nerve root associated with a partial defect of the right neural arch of L5 in a patient who showed radiculopathy without definite lumbar disc herniation. In the present case, radiography revealed spina bifida occulta at the L5 level.

In clinical symptoms, twin dermal involvement even in a single intervertebral lesion has been proposed to be indicative of CNRs because two roots may be compressed by the ruptured discs. Another clinical clue to the suspicion of CNRs is a negative Lasègue sign despite radiculopathy [4]. The present case also showed right leg radiculopathy originating from the S1 and L5 nerve roots, suspected from the distribution of the muscle weakness, as well as from the lumbar disc herniation at the right L5/S1 intervertebral disc level. In contrast, the Lasègue sign was strongly positive at 20° on the right side, even though the CNR was inherently less mobile, that is, impingement or strain were unlikely to occur. One of the causes might be due to the strange shapes of pedicle and lamina, which could even impact vertebral canal and intervertebral foramen although a detailed imaging evaluation has not been done.

Radiological diagnosis remains challenging despite improvements in the imaging modalities. MRI is the gold standard for differentiating CNRs from other space-occupying processes. Especially, T2-weighted coronal MR images exactly describe the course of each nerve root [17]. MR neurography is also effective because it enhances selective multiplanar visualization of neural tissue and pathology by encompassing a combination of two-dimensional, three-dimensional, and diffusion imaging pulse sequences [18]. However, even in MR neurography, it is difficult to demonstrate the CNR when a large disc herniation or severe stenosis is present in the spinal canal. Additional conventional myelography will be helpful if MRI cannot be performed or does not offer reliable results [14]. Kang *et al*. [19] reported the “sagittal shoulder sign,” defined as a vertical structure connecting two consecutive nerve roots and the overlying herniated disc on parasagittal MRI. Additionally, Song *et al*. [20] described three radiological signs on standard axial MRI at the level of discs: “corner sign” described as an asymmetric structure of the anterolateral corner of the dural sac with one side being angulated compared to the other, “fat crescent sign” that refers to the presence of extradural fat between the CNR and the asymmetric dural sac, and “parallel sign” that denotes an unusual course of the entire nerve root at the disc level, running parallel to the disc plane. In the present case, a CNR could not be indicated in conventional myelography because sufficient description by contrast medium was not achieved. However, only the laterality of the thickness and exit angle of the conjoined L5 and S1 nerve roots that was identified as Type 1A in T2 coronal MR images and MR neurography was retrospectively confirmed although specific signs on MRI were lacking.

The operative management of NRAs depends on neurological problems and the clinical conditions of each individual case. Asymptomatic and incidentally diagnosed cases do not require treatment. The presence of NRAs may affect the success of discectomy [21]. Congenital NRAs of the lumbosacral spine that are unexpectedly encountered during surgery for lumbar disc herniation could be mistaken for a proportion of protruding or herniated disc and incised inadvertently, resulting in iatrogenic neural injury [1]. Cannon *et al*. [2] reported unsatisfactory results with hemilaminectomy and discectomy in patients with lumbar disc herniation and CNRs. White *et al*. [11] stated that only a 30% success rate may be achieved with a standard discectomy technique, and emphasized the importance of additional pediculectomy to remove the pedicle until it had become flush with the corresponding vertebral body. Furthermore, CNRs are less mobile than the normal roots. Greater root thickness and root exit angle make it much more difficult to retract medially, thus leading to later-onset neuropathy and root injury [21]. Wide exposure by hemilaminectomy allows adequate visualization and mobilization of the involved roots and aids defining the CNRs and their origin, thereby avoiding the risk of laceration and excessive traction [22]. Artico *et al*. [10] stated that it is necessary to keep in mind that a hemilaminectomy with sufficient exposure of the intervertebral foramen or lateral recess should be performed to avoid alterations in stability and to ensure correct mobility of the lumbosacral spine. In our case, a CNR could be confirmed through meticulous exposure by enlarged fenestration under a microscope during surgery. Eventually, the fragments of HNP were completely removed piece by piece from the axillary part of the involved CNR, which was thick and less mobile.

## Conclusions

In summary, correct diagnosis, clinical and prognostic evaluation, surgical planning, and management must be carefully performed in cases of lumbar disc herniation accompanying an NRA represented by CNR. MR neurography, which is extremely useful for the clear visualization of the neural tissue, should be proactively considered in HNP cases accompanied by the bony anomalies of the lumbosacral spine, especially in the lumbosacral lesion to detect an NRA. Discectomy under a microscope, which enables magnified three-dimensional observation of the surgical field must provide a valid and safe procedure to achieve adequate exposure of herniated discs for secure resection and satisfactory alleviation of clinical symptoms.

## Data Availability

All data generated or analyzed during this study are included in this published article.

## Abbreviations

NRA: Nerve root anomaly
CNR: Conjoined nerve root
MMT: Manual muscle test
TA: Tibialis anterior
PL et PB: Peroneal longus et brevis
EHL: Extensor hallucis longus
FHL: Flexor hallucis longus
TS: Triceps surae
CTM: CT following myelography
HNP: Herniated nucleus pulposus
